# Stabilization of a Clayey Soil with Ladle Metallurgy Furnace Slag Fines

**DOI:** 10.3390/ma13194251

**Published:** 2020-09-24

**Authors:** Alexander S. Brand, Punit Singhvi, Ebenezer O. Fanijo, Erol Tutumluer

**Affiliations:** 1 Charles E. Via, Jr. Department of Civil and Environmental Engineering, Virginia Polytechnic Institute and State University, 750 Drillfield Drive, Blacksburg, VA 24061, USA; ebenfanijo@vt.edu; 2 Department of Civil and Environmental Engineering, University of Illinois at Urbana-Champaign, 205 N. Mathews Avenue, Urbana, IL 61801, USA; singhvi3@illinois.edu (P.S.); tutumlue@illinois.edu (E.T.)

**Keywords:** steel furnace slag (SFS), ladle metallurgy furnace (LMF) slag, soil stabilization, unconfined compressive strength, dynamic modulus, slag characterization

## Abstract

The research study described in this paper investigated the potential to use steel furnace slag (SFS) as a stabilizing additive for clayey soils. Even though SFS has limited applications in civil engineering infrastructure due to the formation of deleterious expansion in the presence of water, the free CaO and free MgO contents allow for the SFS to be a potentially suitable candidate for clayey soil stabilization and improvement. In this investigation, a kaolinite clay was stabilized with 10% and 15% ladle metallurgy furnace (LMF) slag fines by weight. This experimental study also included testing of the SFS mixtures with the activator calcium chloride (CaCl_2_), which was hypothesized to accelerate the hydration of the dicalcium silicate phase in the SFS, but the results show that the addition of CaCl_2_ was not found to be effective. Relative to the unmodified clay, the unconfined compressive strength increased by 67% and 91% when 10% and 15% LMF slag were utilized, respectively. Likewise, the dynamic modulus increased by 212% and 221% by adding 10% and 15% LMF slag, respectively. Specifically, the LMF slag fines are posited to primarily contribute to a mechanical rather than chemical stabilization mechanism. Overall, these findings suggest the effective utilization of SFS as a soil stabilization admixture to overcome problems associated with dispersive soils, but further research is required.

## 1. Introduction

Dispersive and soft clayey soils are some of the most problematic soils due to their poor and vulnerable engineering properties [1,2], such as their expansive nature, excessive cracking, low compressive and shear strengths, low modulus, large settlement under loading, high volumetric shrinkage, and poor durability against wetting/drying and freezing/thawing cycles [3], which imposes severe damage to and/or failure of geotechnical structures [4,5,6]. For decades, various industrial-based chemical stabilizers, such as Portland cement, lime, asphalt, and polymers, have been proven to improve the quality of clayey soils [7,8,9], but can be considered to demand high economic and/or environmental costs. The increased cost associated with traditional chemical stabilizers has led researchers to develop alternative soil modifiers from industrial by-products [10,11,12,13,14], such as ground granulated blast furnace slag, fly ash, and cement kiln dust, which provide both economic and environmental solutions to resource conservation in soil engineering. 

Steel furnace slag (SFS) from the steelmaking and steel refining processes is a common by-product produced around the world [15,16,17,18,19]. An estimated 169–254 million tons of SFS were produced worldwide in 2017 [20]. Aside from its abundance, SFS has been regarded as a potential material for use in civil infrastructure applications [16], such as Portland cement concrete, asphalt concrete, road base, ballast, embankment, and soil stabilization. From recent studies, there is limited usage of SFS, particularly in bound applications (e.g., aggregate in Portland cement concrete or in cement-treated bases) due to the deleterious expansion that it undergoes when the free calcium oxide (CaO) and magnesium oxide (MgO) present in the SFS react with water [16,21,22,23,24]. Free CaO and free MgO expand by 92% and 120%, respectively, when reacting with water to form hydroxides [25]. The free CaO content ranges for two types of SFS, which include basic oxygen furnace (BOF) slag and electric arc furnace (EAF) slag, are 1–10% and 0–4%, respectively [19]. This expansion can be extensive and result in structural failures (e.g., failure due to swelling, expansive cracking, loss in strength, etc.) [26,27,28]. 

However, the free CaO and free MgO in SFS can be effectively used in geotechnical engineering applications, such as stabilization of clayey soils and improvement of their engineering properties. Similar to the performance of lime in stabilizing dispersive soils [29,30,31], the cation exchange, flocculation and agglomeration, and pozzolanic reaction can occur as a clayey soil reacts with certain free oxides in SFS. Various research studies have been conducted to employ SFS to enhance the properties of problematic soils, while primarily demonstrating that SFS can be an effective stabilization agent [32,33,34,35,36,37,38,39,40,41,42,43,44,45,46,47,48,49]. From the early 1990s, Akinmusuru [43] pioneered the attempt to use SFS in soil stabilization for rural roads with low traffic volume, which suggests that SFS possesses the potential to improve the soil properties by increasing the strength, California bearing ratio (CBR), and dry density. Subsequent results by Poh et al. [45] showed that using BOF slag for soil improvement was not encouraging. Despite the employment of chemical activators, the engineering properties (dry density, strength, and durability) of BOF-stabilized samples were lower than those properties improved by cement stabilization. A recent study of Akinwumi [42] concluded that the addition of SFS to lateritic soil increased the dry density, decreased the optimum moisture content, and, as the percent SFS increased, the soaked and un-soaked CBR and the unconfined compressive strength increased. Zumrawi and Babikir [47] studied the effectiveness of adding 5%, 10%, 15%, 20%, and 30% of SFS to an expansive soil and reported that the addition of SFS improved soil properties. Abdi [49] investigated stabilizing a kaolinite soil with a combination of BOF slag and hydrated lime, which demonstrated that higher BOF slag with lime contents yielded higher compressive strengths. This finding also agreed with the work of Yildirim et al. [44], which evaluated soil stabilization with the blend of SFS and either Class C fly ash or ground granulated blast furnace slag. Manso et al. [36] found that clay soils stabilized with ladle furnace slag could have bearing capacities similar to lime-stabilized clay soils.

One major concern in using SFS, relative to more traditional stabilizing materials, is the slow rate of hydration at early stages due to the low activity of calcium silicates in SFS [50]. Some studies have attempted to accelerate the hydration of SFS by adding a chemical activator such as quicklime, sodium hydroxide (NaOH), calcium chloride (CaCl_2_), sodium chloride (NaCl), or sodium metasilicate pentahydrate (Na_2_SiO_3_-5H_2_O) [50]. Although studies have shown that quicklime and Na_2_SiO_3_-5H_2_O were effective in accelerating the hydration of the SFS for soil stabilization [45], CaCl_2_ was found to be more effective to increase the SFS hydration rate both not only for soil improvement but also in SFS paste and as a replacement in concrete [51].

Therefore, this study focuses on evaluating the use of SFS for improving the engineering properties of a clayey soil. The clayey soil selected has a low bearing capacity, which makes it unsuitable for any road base, foundation, and other construction projects. The soil stabilization was carried out at 10% and 15% additions of SFS by weight. Ladle metallurgy furnace (LMF) slag, also known as secondary refining slag, was employed in this study, which is a promising SFS type and shares common chemical features with other steel slags [18,21,36,52,53,54,55]. In addition, CaCl_2_ was selected as a potential chemical admixture to accelerate the hydration of the dicalcium silicate phase in the SFS. The primary contribution of the research is the use of LMF slag for clayey soil stabilization, which has seen little focus in the literature (e.g., References [36,38] used LMF slag powder), and particularly LMF slag fines, which have not been used in the literature as a soil stabilizer and which offer potential for both chemical and mechanical stabilization mechanisms.

### Overview of SFS Selection and Properties

SFS is a by-product generated during the steelmaking process. Most SFS are generally classified as either by the conversion of iron to steel in a basic oxygen furnace (BOF) or the melting of scrap to make steel in an electric arc furnace (EAF). In fact, 99.6% of the steel produced worldwide is produced by either the BOF or the EAF process [56], with around 71% of the worldwide production using the BOF process [56]. Further refinement of the steel during secondary steelmaking can occur after the BOF or EAF processes from which ladle metallurgy furnace slag (LMF) is produced. For BOF production, liquid blast furnace metal, scrap, and various fluxes, consisting of lime or dolomitic lime, are charged to the furnace, and high-pressure oxygen is injected through a lance. The impurities, which include carbon monoxide, silicon, manganese, phosphorus, and liquid oxides, combine with the lime or dolomitic lime to form the slag. The EAF process is different in that it electrically charges cold steel scraps such as iron scrap, pig iron, and direct reduced iron. The steel scrap is melted with the charge and brought up to the required chemical composition by adding other metals. Oxygen is then blown into the EAF to purify the steel, which creates a slag layer that will float on the top and can be poured off. 

The chemical composition of SFS may vary by plant and even by batch. As a byproduct of steel production, it is dependent on the raw materials, types of steel produced, furnace conditions, cooling processes, etc. [15,16]. The primary components in most SFS are oxides of calcium, magnesium, aluminum, silicon, and iron [15,16]. Mineralogically, BOF slags consist by weight mainly of 30–60% dicalcium silicate (2CaO-SiO_2_), 0–30% tricalcium silicate (3CaO-SiO_2_), 0–10% free CaO, 10–40% wüstite (FeO), and 5–20% dicalcium ferrite (2CaO-Fe_2_O_3_) [19]. Comparatively, Portland cement is composed of about 55% tricalcium silicate (3CaO-SiO_2_), 18% dicalcium silicate (2CaO-SiO_2_), 10% tricalcium aluminate (3CaO-Al_2_O_3_), 8% tetracalcium aluminoferrite (4CaO-Al_2_O_3_-Fe_2_O_3_), and 6% gypsum (CaSO_4_-2H_2_O) [57]. Therefore, there is potential for SFS to behave as a slow-reacting cementitious material [58,59,60,61,62].

## 2. Materials and Methods 

### 2.1. Material Selection 

A clayey soil with a low bearing capacity was selected for this experiment. The soil and the LMF slag were characterized using several methods. The gradations of both the LMF and the clayey soil were evaluated by ASTM C136 while the LMF slag specific gravity and absorption were characterized by ASTM C128. ASTM C29 was used to evaluate the LMF unit weight (rodding method). Additionally, the soil for this stabilization project was a refractory clay, which is commonly utilized to make ceramics but is useful in soil studies for its plastic properties. The plastic limit (PL), liquid limit (LL), and plasticity index (PI) were determined according to ASTM D4318.

For this experimental study, the SFS provided for stabilization was a ladle metallurgy furnace (LMF) slag, which was produced by a modified EAF process. The LMF process can introduce more free lime (CaO) in the slag than the typical EAF process. The LMF slag was provided by the Edw. C. Levy Co. from a plant in Crawfordsville, IN, USA. 

### 2.2. Chemical and Mineralogical Characterization 

Mineralogical characterization of the LMF slag and the clayey soil was conducted using powder X-ray diffraction (XRD). The LMF slag was crushed to a particle size passing the No. 100 sieve (≤150 μm). The material passing the No. 200 sieve (≤75 μm) was used to determine the mineralogy of the clayey soil. A Siemens-Bruker D5000 XRD (Bruker, Billerica, MA, USA) was used, which utilizes copper (Cu) Kα radiation and has a graphite monochromator and a scintillation detector. The machine was operated at 40 kV and 30 mA. The sample size was 0.5 cm^3^ (0.03 in^3^). The 2*θ* scan range was from 10° to 80° with an increment of 0.02° and a scan speed of 0.5°/min.

Likewise, additional quantification of the LMF slag composition was conducted using thermogravimetric analysis (TGA), which has been commonly utilized to better assess the total calcium oxide (CaO) and calcium hydroxide (Ca(OH)_2_) contents present in SFS, based on the method proposed by Brand and Roesler [21]. In this study, a TA Instruments Q50 TGA (TA Instruments, New Castle, DE, USA) was utilized, which heated the sample to 1000 °C at a heating rate of 10 °C per minute to derive the weight loss. Nitrogen was used as the purge gas at flow rates of 60 mL/min for the sample purge and 40 mL/min for the balance purge.

Complexometric titration was utilized to determine the free lime content. In this technique, a sample of SFS is mixed with hot ethylene glycol, filtered, and then titrated with acid after an indicator has been added. Ethylene glycol extraction methods were originally developed to rapidly determine the free lime content of Portland cement and clinker [63], but have since been adopted for SFS (e.g., References [64,65,66,67]). Specifically, the method from Brand and Roesler [21,68] was followed: about 1 g of SFS passing the No. 100 sieve (≤150 μm) was weighed and continuously stirred with 50 mL of ethylene glycol in a water bath at 95 ± 5 °C for 30 min. After filtering, 10 drops of a phenolphthalein indicator were added and then titrated with 0.1 N hydrochloric acid (HCl). The “ethylene glycol number” (EGN) is calculated as follows based on the initial mass of the SFS sample (m), the normality of the HCl (*N_HCl_*), the volume of HCl titrated (*V_HCl_*), a correction for the volume of HCl titrated in a blank ethylene glycol sample (*V_blank_*), and an equivalency factor (*F*).

(1) $$\mathrm{EGN}=F\left[\frac{N_{HCl}\left(V_{HCl}-V_{blank}\right)}{10\;m}\right]$$

The correction factor *F* for this method is 28 [21,69,70]. The correction *V_blank_* is specified in other standards [71] to account for the amount of HCl needed to titrate a plain solvent sample (i.e., plain ethylene glycol). It was found that *V_blank_* = 0 mL, which is a reasonable result since the pH of ethylene glycol is close to neutral. The EGN value accounts for the available Ca^2+^ ions from the free CaO and Ca(OH)_2_, so the free lime content needs to be adjusted based on the Ca(OH)_2_ determined by TGA [21].

Furthermore, the experimental design consisted of five total mixtures to be compacted at optimum moisture content, including the unmodified and SFS-stabilized clay. Moisture-density relationships were conducted to determine the optimum moisture content for the unmodified clay and the two SFS content mixtures (10% and 15% by weight). Based on the literature review, it was deemed that calcium chloride (CaCl_2_) may act as a suitable accelerator for the dicalcium silicate phase in the SFS. Therefore, additional mixtures at 10% and 15% SFS were made with 2% CaCl_2_ (by weight of total water). All five mixtures were then tested for unconfined compressive strength and dynamic modulus.

### 2.3. Moisture-Density Relationships 

The standard Proctor test according to ASTM D698 was employed to determine the moisture-density relationship for unmodified clay and the clay samples modified with 10% and 15% SFS by weight. The materials were compacted at different moisture contents until the maximum dry density was achieved. The mixing was carried out using a mechanical mixer to accomplish uniform mixing. The compacted samples were weighed and recovered for determining actual moisture content. The optimum moisture content corresponding to maximum dry density was then estimated using the moisture density curves.

### 2.4. Unconfined Compressive Strength (UCS)

To evaluate the suitability of using the SFS modified mixtures in subgrade stabilization, it is important to quantify the effects of these stabilizers on the sample strength gain characteristics in comparison to unmodified clay strength properties. For this purpose, an unconfined compressive test for the unmodified clay was carried out using ASTM D2166, which was followed by testing the SFS-modified mixtures using ASTM D5102. This is for determining the UCS of compacted soil-lime mixtures.

The mold used for preparing the samples was 2.8 inches (7 cm) in diameter with a height of 5.6 inches (14 cm) with a diameter to height ratio of 1:2. The samples were compacted in the mold at the optimum moisture content to achieve the maximum dry density. The maximum dry density and optimum moisture content information was also employed to determine the exact weight of clay, SFS, and water to result in a specimen of required dimensions as mentioned above. The specimen was compacted into three equal layers. 

The unmodified sample was tested immediately after compaction, whereas the modified specimens were wrapped in plastic to avoid moisture loss and then subjected to accelerated curing at 49 °C for 48 h. The accelerated curing used for this research has been tested as equivalent to the 28-day strength of soil-lime mixtures at 23 °C [72,73]. The cured samples were tested for unconfined compressive strength at a displacement rate of 1.0 mm/min. The test performed was stress controlled. The peak load measured was recorded as the unconfined compressive strength.

### 2.5. Vibration Resonance 

In addition to the UCS testing, the improvement to the properties of the soil with the addition of SFS was also investigated by studying the dynamic modulus of the compacted soil. One such method of measuring the dynamic modulus is by vibration resonance. An impact event, when incident on a specimen of finite size, will generate various waves in the specimen, namely primary, secondary, and surface waves. The multiple reflections of the primary and secondary waves will eventually set up a vibration resonance in the sample, which is a function of the dynamic modulus and density of that material [74]. Since the vibration resonance acts to “homogenize” the specimen, the test method can be applied to heterogeneous materials to determine the dynamic modulus, provided that the size of the specimen is larger than the constituents. The dynamic modulus (*E_d_*) can be computed based on the density (*ρ*), length (*L*), and fundamental longitudinal frequency (*f_l_*) of the specimen as follows.

(2) $$E_{d}=\rho {\left(2f_{l}L\right)}^{2}$$

Vibration resonance testing, while more commonly applied to concrete materials according to ASTM C215, has been applied to both stabilized and un-stabilized soils [75,76,77,78,79,80]. Guimond-Barrett et al. [75] found repeatable resonance tests between multiple specimens of soil-cement mixtures, which indicates that it is useful for such heterogeneous materials as stabilized soils. A good agreement was reached between the resonant frequency dynamic modulus measured in the laboratory and the modulus measured in the field [76]. In addition, Hilbrich and Scullion [77] determined that a reasonable agreement existed between the resonant frequency dynamic modulus and the resilient modulus of stabilized soils. 

**Figure 1:**
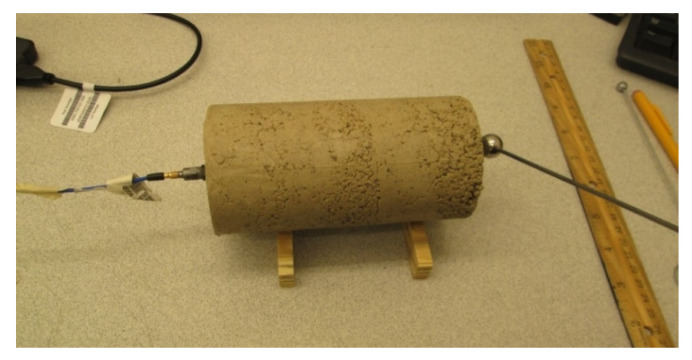
Cylindrical specimen and configuration for testing the longitudinal resonance frequency.

In this laboratory experiment, a compacted cylindrical specimen for the unstabilized and stabilized clay mixtures were tested for longitudinal resonance using three impactor sizes (8, 14, and 18 mm). The experiment configuration followed ASTM C215 for the support arrangement, impact location, and accelerometer location (Figure 1). The accelerometer voltage and time were recorded and post-processed by a fast Fourier transform (FFT) algorithm and plotted in the frequency domain to determine the resonance frequency. For each signal, 50,000 data points were collected with a sample interval of 2 μs for a spectral line spacing of 10 Hz.

## 3. Results and Discussion 

### 3.1. Characterization of LMF Slag 

In accordance with standards, the averages of three replicate gradation tests performed on the LMF slag fines and the fine clayey soil are illustrated in Table 1. The standard deviation was ≤2.3% for the replicate gradation measurements. The LMF slag had 100% passing the No. 4 (4.75 mm) sieve with about 11% passing the No. 200 (75 μm) sieve. The clayey soil had about 15% passing the No. 200 (75 μm) sieve. Table 1 indicates that the clayey soil had a finer gradation than the LMF slag, as was expected. Additionally, the LL, PL, and PI results of the clayey soil are summarized in Table 2. With about 15% of the material passing the No. 200 (75 μm) sieve, the clayey soil is characterized as an AASHTO A-2-6 soil.

Table 3 displays the specific gravity (G_S_) and absorption of the LMF slag fines, as the average of three replicate tests. Oven dry (OD) and saturated surface dry (SSD) moisture conditions were assessed for the relative G_S_. Typically, steel slags have higher G_S_ values due to the presence of iron content. The specific gravity of SFS aggregates can be around 3.2 to 3.5 [81]. The absorption was relatively high for the LMF slag fines due to production and processing. Typical SFS absorption values have been reported to be around 0.2% to 1.0% [82]. 

Mineralogical characterizations of the LMF slag and the clayey soil using XRD are summarized in Table 4. Qualitative analysis of the XRD revealed the presence of wüstite (FeO), larnite (β-dicalcium silicate, Ca_2_SiO_4_), mayenite (Ca_12_Al_14_O_33_), and periclase (MgO) in the LMF slag. Recent research by Brand and Roesler [21,55] also revealed that larnite and wüstite are common mineral phases in any steel slag type (EAF, BOF, and LMF slags), which is also consistent with Motz and Geiseler [17]. While phases like mayenite and periclase or magnesium oxide (MgO) are mostly seen in EAF slags, the LMF slag also show only the presence of both oxides [18,83]. The clayey soil was also characterized by XRD. It was indicated that the soil primarily consisted of quartz (SiO_2_) and kaolinite (Al_2_Si_2_O_5_(OH)_4_).

**Figure 2:**
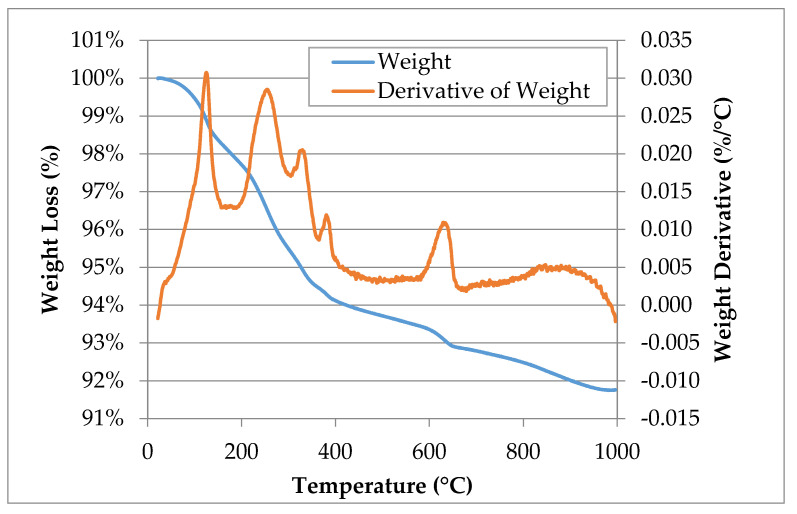
TGA result of the LMF slag, which indicates the weight loss and the derivative of the weight loss.

Table 5 lists the corresponding identified phases while Figure 2 illustrates the graphical representation of the TGA result of SFS. The total content of a given phase was determined stoichiometrically based on weight loss. The identified mass loss phases included free and chemically-bound water, magnesium hydroxide (Mg(OH)_2_), calcium hydroxide (Ca(OH)_2_), and calcium carbonate (CaCO_3_). From Figure 2, the decomposition of Mg(OH)_2_, Ca(OH)_2_, and CaCO_3_ lies at an approximate temperature of 330 °C, 400 °C, and 600–650 °C, respectively, which agree with the ranges reported in the literature [21,66,84].

The result of the complexometric titration indicated that the free lime content of the LMF slag was about 2.5% (Table 6)). Coupled with the 1.3% Ca(OH)_2_, 3.2% Mg(OH)_2_, and an unknown amount of free MgO, there are multiple phases in this LMF slag sample that can contribute to soil stabilization mechanisms.

### 3.2. Moisture-Density Relationships 

**Figure 3:**
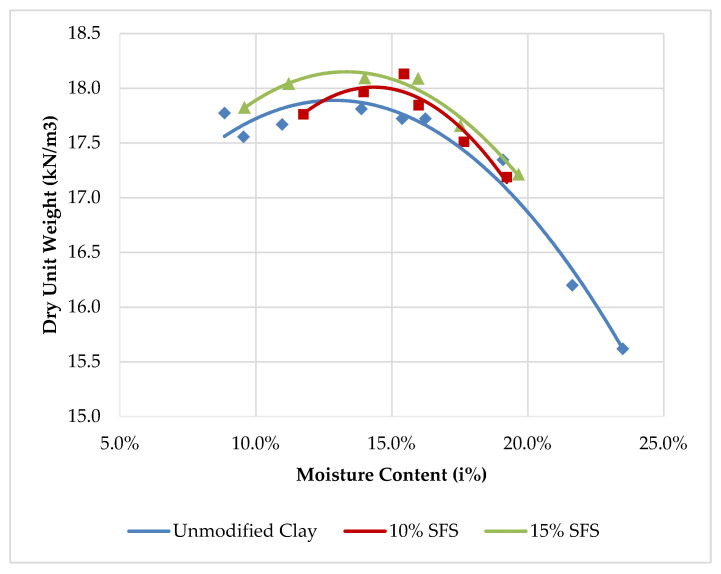
Moisture-density relationships for unmodified clay, and clay modified with 10% SFS, and 15% SFS.

The standard Proctor test was used to determine the moisture density relationship for unmodified clay and clays modified with 10% SFS and 15% SFS by weight of the total mix. Figure 3 shows the moisture-density results of the different clay samples. Table 7 summarizes the optimum moisture contents and corresponding maximum dry densities for the unmodified and the SFS-modified clay samples. The values suggest that, as the amount of SFS is increased, the optimum moisture to achieve the maximum dry density is also increased possibly as a result of the high absorption capacity of the SFS. In addition, the maximum dry density was also observed to increase with growing SFS content, which is likely due to the SFS having a higher specific gravity than the clay. 

### 3.3. Unconfined Compressive Strength (UCS)

Three replicate UCS tests were performed for each of the five mixes. The unmodified clay samples were tested immediately after compaction. It was assumed that the addition of the CaCl_2_ did not affect the optimum moisture content, so these specimens were mixed at the optimum moisture content determined for the SFS mixtures without CaCl_2_. The SFS-modified samples and SFS-modified samples with CaCl_2_ were tested after curing for 48 h at 49 °C. An increase in average UCS was observed with growing SFS content. However, the addition of CaCl_2_ in SFS-modified clay samples showed a reduction in the UCS compared to the equivalent SFS-modified samples without CaCl_2_, which was also shown by Thomas [85]. Meanwhile, the UCS for SFS modified clay with CaCl_2_ was greater than unmodified clay, as observed in the work of Poh et al. [45]. 

**Figure 4:**
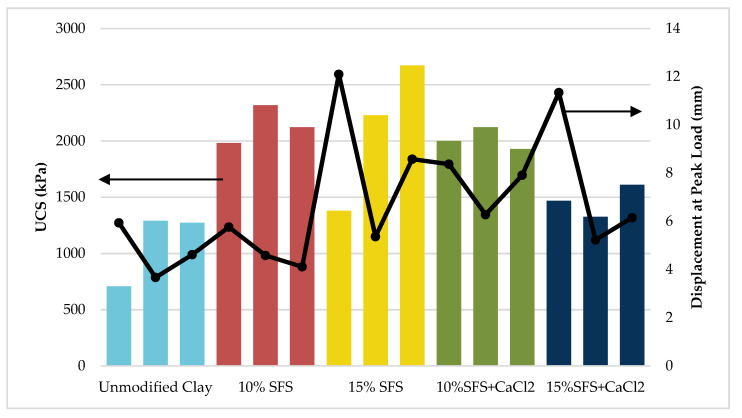
Unconfined compressive strength (UCS) comparison of clay samples.

**Figure 5:**
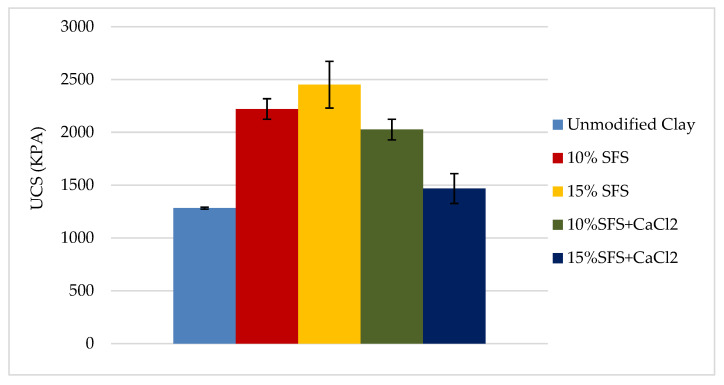
Average UCS for each mixture. Error bars indicate one standard deviation.

Figure 4 shows the comparison of three replicates tested for each mixture. Table 8 summarizes the values measured for UCS for different mixtures. The results indicate that there was variability in UCS and the displacement at peak load in the three replicates. However, the displacement at peak load was not necessarily differentiated between the unmodified and SFS-modified clay samples. The average UCS for each mixture is shown in Figure 5, which indicates that the SFS-modified clay samples had greater standard deviations than the unmodified clay. This suggests that there was greater variability in the chemical reactions and/or compaction. 

Despite accounting for the moisture content of the clay and SFS, it can be observed in Table 8 that the actual moisture at compaction was higher than the optimum moisture content. This can contribute to the variability in the findings since not all samples were, therefore, compacted to maximum density [33]. 

**Figure 6:**
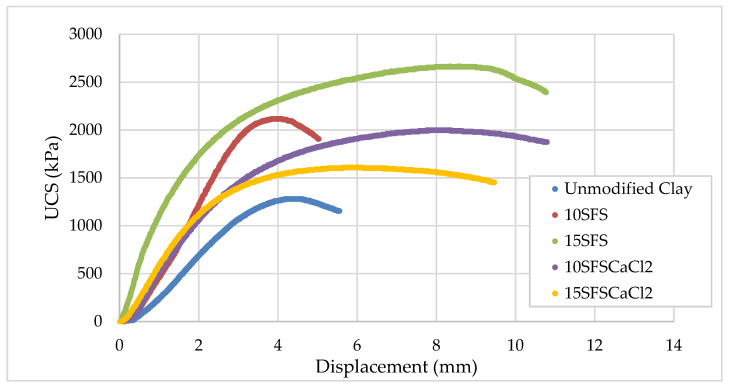
Average stress-displacement curve for each mixture.

**Figure 7:**
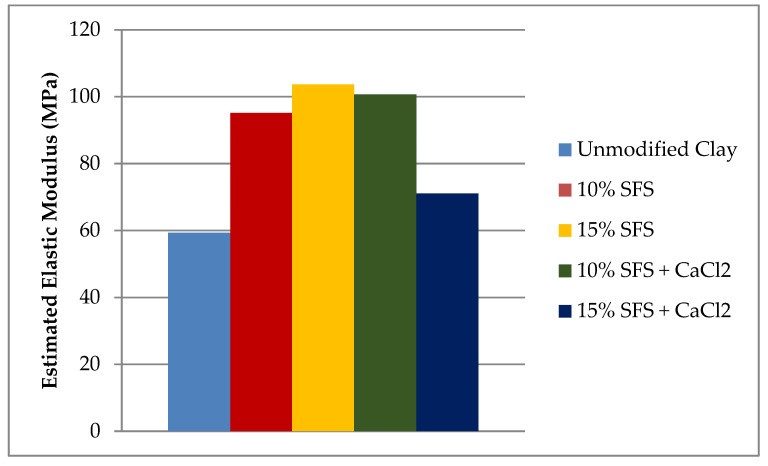
Average estimated elastic modulus for each mixture.

The stress-displacement curves for the various mixtures and replicates are shown in the Supplementary Material (Figure S1), with averages compared in Figure 6. The stress-displacement behavior suggests that the modulus of the stabilized clay samples is greater than the unmodified clay. Estimating the elastic modulus based on the linear portion of the stress-strain curves reveals that the elastic modulus for the different mixes follows the same trend as with UCS, as demonstrated in Figure 7. However, it is noted that the CaCl_2_ addition did not affect the elastic modulus relative to the 10% SFS mix, but the limited dataset for the 10% SFS mix may skew this finding. The addition of 10% SFS increased the elastic modulus by 60% while 15% SFS increased the elastic modulus by 75%, relative to the unmodified clay.

### 3.4. Vibration Resonance

For the unmodified clay, the longitudinal resonant frequency was found to be more repeatable (coefficient of variation of 1.3%) when compared to the transverse resonant frequency (coefficient of variation of 3.1%), as can be seen in Table 9. The larger impactors generated a transverse resonant frequency that was lower than that generated by the small impactor, which is unexpected since the resonant frequency is a fundamental material property and should not change. This finding suggests that the unmodified clay has a damping effect that influences the resonant frequency. The possible damping effect of the clay may have been a factor for previous studies that determined the longitudinal resonant frequency [75,76,77]. Comparing the impactor size (see Supplementary Material, Figure S2), it is evident that the small impactor induced the most prominent response. The larger impactors additionally induced some noise in the signal before the resonant frequency for both the longitudinal and transverse testing. 

**Figure 8:**
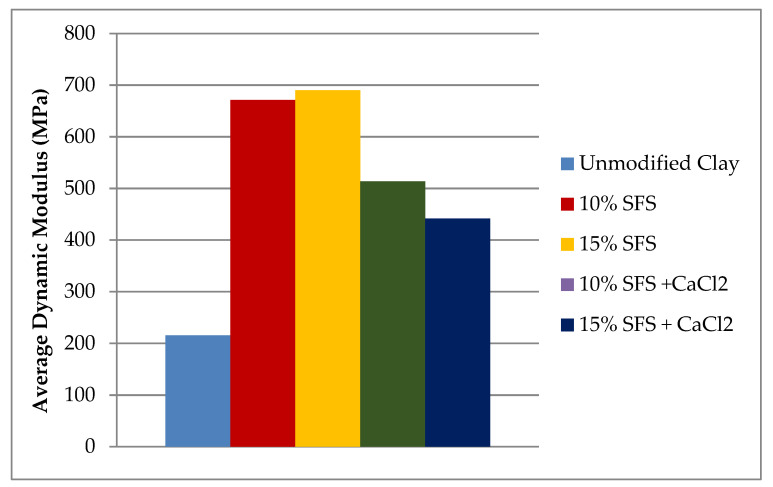
Comparison of the dynamic modulus generated by the 8-mm impactor for each mix.

Given the higher variability in the transverse resonant frequency, only the longitudinal resonant frequency was determined for the stabilized mixtures along with the corresponding dynamic modulus (Table 10). As shown in Table 10, the longitudinal resonant frequency was higher for the stabilized mixtures when compared to the unmodified clay, which resulted in higher dynamic moduli for the stabilized mixtures (Figure 8). Relative to the unmodified clay, the increases in dynamic modulus were 212%, 221%, 139%, and 105% for the stabilized mixes with 10% SFS, 15% SFS, 10% SFS with CaCl_2_, and 15% SFS with CaCl_2_, respectively. Comparing the stabilized mixes with and without CaCl_2_, the CaCl_2_ was not effective at accelerating the hydration or the reaction of the SFS. 

Note that the dynamic modulus from resonance testing was significantly greater than the estimated elastic modulus from the UCS tests. This is an expected outcome, as has been demonstrated for various geomaterials [86,87,88,89,90] because of differences in strain rate, material heterogeneity and the volume probed, anisotropic effects, stress history, and strain amplitude. For instance, the static elastic modulus testing in this study involved large strains relative to the comparatively small strains in resonant frequency dynamic modulus testing. It was found that the dynamic modulus was 2.5–6 times greater than the estimated elastic modulus.

### 3.5. Effect of SFS Content

A general trend of increasing UCS and dynamic modulus was noted with increasing SFS content, which suggests that: (1) the CaO and Ca(OH)_2_ content of the SFS is reacting with the clay and/or (2) the SFS particles are physically acting to stabilize the soil structure. Certainly the chemical reaction between lime and clay minerals will enhance the engineering properties of the soil [91,92]. However, the total CaO and Ca(OH)_2_ content of the LMF slag was about 3.5%, which may not be sufficient to be the sole cause of the increase in strength and modulus. Therefore, it is likely that the SFS also contributes a mechanical stabilization or modification of the soil, as has been demonstrated in the literature using other aggregates [93,94,95,96]. 

**Figure 9:**
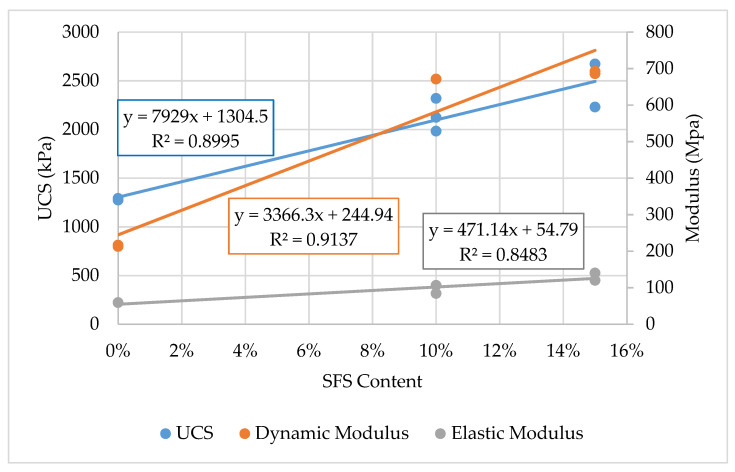
Relationships between UCS and modulus with the SFS content (without CaCl_2_).

Figure 9 also demonstrates that the dynamic and elastic (static) moduli increased with increasing SFS content, with the dynamic modulus increasing more than the elastic modulus. As discussed in Section 3.4, the dynamic and elastic moduli are not equivalent, as demonstrated in other studies [86,87,88,89,90], with the dynamic modulus often being a greater magnitude than the elastic modulus [86]. Therefore, the moduli behavior in Figure 9 is in agreement with the literature. 

As shown in Figure 9, the dataset suggests that a linear trend matches the increase in properties at least up to 15% SFS. However, the dataset is too limited to definitively assess and characterize the relationship between the SFS content and the hardened properties of the stabilized clay. 

### 3.6. Effect of CaCl_2_ Addition

Relative to the SFS-modified clay samples, the addition of the CaCl_2_ reduced the UCS and dynamic modulus of the stabilized clay. With 10% SFS, the addition of CaCl_2_ reduced the dynamic modulus by 23% while, with 15% SFS, the reduction was 36% when CaCl_2_ was added. Taylor [97] reports that the accelerating effect of CaCl_2_ is more pronounced for Portland cement at lower temperatures. Therefore, perhaps, the elevated curing temperature adversely affected the accelerating ability of the CaCl_2_. In addition, while the use of chloride salts appears to enhance the reaction of lime with clayey soils [98,99,100], it is possible that the elevated curing temperature accelerated the carbonation of the free CaO in the SFS [101], which could have, therefore, negated the effectiveness of chloride in accelerating the lime-clay reaction. It has also been reported that the calcium silicate phase(s) in SFS are poorly reactive or relatively inert [81,102], which would additionally suggest that the CaCl_2_ did not sufficiently affect the SFS reactivity.

## 4. Conclusions

The study was undertaken to investigate the potential for stabilizing a clayey soil with steel furnace slag (SFS). The SFS employed was specifically a finely graded ladle metallurgy furnace (LMF) slag. The LMF slag was found to be composed of wüstite, larnite (β-dicalcium silicate), mayenite, and periclase with approximately 2.5% free lime, 1.3% calcium hydroxide, and 3.2% magnesium hydroxide. The soil, selected for its plastic properties, was a refractory clay composed of quartz and kaolinite and was classified as an AASHTO A-2-6 soil with a plasticity index of 14. 

Two SFS contents were tested, which included 10% and 15% LMF slag by weight. The moisture-density relationships revealed that the maximum dry density and optimum moisture content increased with increasing SFS content. The results indicated a linear trend for increasing unconfined compressive strength (UCS) and dynamic modulus with increasing SFS content. Relative to the unmodified clay, the UCS increased by 67% and 91% when 10% and 15% SFS were utilized, respectively. The elastic modulus increased by 60% and 75% when 10% and 15% SFS were used, respectively, and the dynamic modulus increased by 212% and 221% when 10% and 15% SFS were added, respectively. Based on the literature, additional SFS modified samples were created with calcium chloride added at 2% by weight of the total water in an attempt to accelerate the hydration of the dicalcium silicate in the LMF slag, but the results suggested that calcium chloride was not effective. 

These findings suggest that LMF slag fines are suitable for stabilizing clayey soils. While there was insufficient evidence of a chemical stabilization mechanism, it is likely that the SFS at least contributed to a mechanical stabilization mechanism. The results also indicate that 15% SFS provides the most improvement to the UCS and dynamic modulus of the stabilized soil, even though further testing is required to validate and improve upon these findings. 

## Figures and Tables

**Table 1:** Average gradations of the LMF slag and clayey soil.

Sieve Size		Average Cumulative Percent Passing	
US	mm	LMF Slag	Clayey Soil
1/4”	6.35	100.0	100.0
#4	4.75	100.0	100.0
#8	2.36	99.9	97.9
#16	1.18	89.2	83.1
#30	0.6	60.4	66.9
#50	0.3	38.2	42.8
#100	0.15	21.9	28.8
#200	0.075	10.8	14.6

**Table 2:** Clayey soil physical properties.

Liquid Limit	Plastic Limit	Plasticity Index	Percent Passing #200 Sieve
35	21	14	14.6%

**Table 3:** Average G_S_ and absorption values for the LMF slag fines.

	Relative G_S_ (OD)	Relative G_S_ (SSD)	Apparent G_S_	Absorption (%)
Average value	2.575	2.767	3.186	7.46%
Standard deviation	0.018	0.017	0.018	0.09%

**Table 4:** Mineral phases identified by qualitative XRD.

Mineral Phases	LMF Slag	Fine Clayey Soil
Kaolinite, Al_2_Si_2_O_5_(OH)_4_		X
Quartz, SiO_2_		X
Larnite, β-dicalcium silicate, Ca_2_SiO_4_	X	
Periclase, MgO	X	
Mayenite, Ca_12_Al_14_O_33_	X	
Wüstite, FeO	X	

**Table 5:** Phase identification and content by TGA.

Identified Phases	Peak Decomposition (°C)	Mass Loss Range (°C)	Mass Loss	Phase Content
Free water	126.6	89–155	1.33%	1.33%
Chemically bound water	255.6	190–302	2.39%	2.39%
Mg(OH)_2_	332.7	302–362	0.98%	3.18%
Ca(OH)_2_	380.7	362–395	0.32%	1.32%
CaCO_3_	629.0	580–660	0.57%	1.29%

**Table 6:** Free lime content of the LMF slag fines.

EGN Value from Titration (%)	Ca(OH)_2_ Content from TGA (%)	Stoichiometric CaO Content in Ca(OH)_2_ (%)	Estimated Free CaO Content (%)
3.45	1.32	1.00	2.45

**Table 7:** Optimum moisture content and maximum dry density values for the unmodified and SFS-modified clay.

Mixture Type	Optimum Moisture Content (%)	Maximum Dry Density (kg/m^3^)
Unmodified Clay	13.70%	17.82
10% SFS	14.25%	18.01
15% SFS	15.00%	18.10

**Table 8:** Values of unconfined compressive strength (UCS), actual moisture, target moisture, and displacement at peak load.

Mix	Optimum Moisture Content (%)	Actual Moisture Content (%)	Replicateno.	Peak Load (kN)	UCS (kPa)	Displacement at Peak Load (mm)
Unmodified Clay	13.70	15.53	1	0.40 *	707.4	5.94
			2	0.73	1291.4	3.67
			3	0.72	1274.2	4.62
10% SFS	14.25	18.46	1	1.12	1981.6	5.77
			2	1.31	2318.0	4.58
			3	1.20	2122.9	4.12
15% SFS	15.00	18.71	1	0.78 *	1380.3	12.10
			2	1.26	2229.1	5.37
			3	1.51	2671.7	8.58
10% SFS + CaCl_2_	14.25	17.48	1	1.13	1585.8	8.37
			2	1.20	2122.9	6.28
			3	1.09	1928.5	7.92
15% SFS + CaCl_2_	15.00	18.20	1	0.83	1468.6	11.34
			2	0.75	1327.2	5.23
			3	0.91	1609.9	6.15

* Data point considered an outlier and not included in the average.

**Table 9:** Longitudinal and transverse resonant frequencies for the unmodified clay.

Impactor Size	Test Replicate	Transverse Resonant Frequency (Hz)	Longitudinal Resonant Frequency (Hz)
8 mm	1	660	1130
	2	670	1140
	3	620	1140
14 mm	1	660	1130
	2	630	1110
	3	650	1100
18 mm	1	630	1120
	2	620	1140
	3	620	1130

**Table 10:** Longitudinal dynamic modulus for each mixture.

Sample	Impactor Size	Length (cm)	Density (kg/m^3^)	Average Longitudinal Resonant Frequency (Hz)	Average Dynamic Modulus (MPa)
Unmodified Clay	8 mm	14.2	2064.0	1136.7	215
	14 mm	14.2	2064.0	1113.3	206
	18 mm	14.2	2064.0	1130.0	213
10% SFS	8 mm	14.2	2101.2	1990.0	671
	14 mm	14.2	2101.2	1950.0	644
	18 mm	14.2	2101.2	1966.7	656
15% SFS	8 mm	14.2	2146.4	1996.7	690
	14 mm	14.2	2146.4	2025.0	710
	18 mm	14.2	2146.4	2015.0	703
10% SFS + CaCl_2_	8 mm	14.1	2097.6	1755.0	514
	14 mm	14.1	2097.6	1765.0	520
	18 mm	14.1	2097.6	1753.3	513
15% SFS + CaCl_2_	8 mm	14.1	2141.9	1610.0	442
	14 mm	14.1	2141.9	1625.0	450
	18 mm	14.1	2141.9	1593.3	432

## Supplementary Materials

The following are available online at https://www.mdpi.com/1996-1944/13/19/4251/s1. Figure S1: Stress-displacement curves for the five mixtures and replicates. Figure S2: Longitudinal resonance for the un-stabilized clay for each impactor size. Figure S3: Comparison of the longitudinal resonant frequency responses generated with 8-mm impactor for each mix. Table S1: UCS and elastic modulus data for each replicate. Table S2: Replicate test data for longitudinal resonance frequency. Table S3: Replicate test data for transverse resonance frequency. Table S4: Moisture-density data from Proctor tests.
