# Supplementary material for: Stabilization of a Clayey Soil with Ladle Metallurgy Furnace Slag Fines
Source: Materials (Basel). 2020 Sep 24;13(19):4251. doi: 10.3390/ma13194251 (PMC7579171; doi:10.3390/ma13194251)
Supplement: Supplementary file 1 [file materials-13-04251-s001.pdf]

materials

Supplementary

Stabilization of a Clayey Soil with Ladle Metallurgy Furnace Slag Fines

A.S. Brand, P. Singhvi, E.O. Fanijo and E. Tutumluer

Table S1 shows the unconfined compressive strength (UCS) and the estimated elastic modulus for replicate tests, while Figure S1 shows the unconfined compressive strength (UCS) load-displacement curves for the five mixes and replicate tests.

Figure S2 shows the effect of impactor size on the longitudinal resonant frequency, and Figure S3 shows the resonant frequencies per mix. Tables S2 and S3 show the replicate testing data for the longitudinal and transverse resonance frequency. Based on the better repeatability in the longitudinal resonance frequency relative to the transverse resonance frequency across impactor sizes for the unmodified clay sample, only the dynamic modulus based on longitudinal resonance frequency was tested and reported.

Table S4 show the results from Proctor tests for the unmodified clay and for the mixes with 10% and 15% SFS. These data were used to determine the optimum moisture content and maximum dry density.

Table S1. UCS and elastic modulus data for each replicate.

Mix	Replicate no.	Peak Load (kN)	UCS (kPa)	Estimated Elastic Modulus (MPa)
Unmodified Clay	1	0.40*	707.4	N/A**
	2	0.73	1291.4	N/A**
	3	0.72	1274.2	59.3
10% Steel furnace slag (SFS)	1	1.12	1981.6	84.1
	2	1.31	2318.0	N/A**
	3	1.20	2122.9	106.2
15% SFS	1	0.78*	1380.3	51.0
	2	1.26	2229.1	120.0
	3	1.51	2671.7	140.0
10% SFS + $CaCl_2$	1	1.13	1585.8	86.2
	2	1.20	2122.9	115.8
	3	1.09	1928.5	100.0
15% SFS + $CaCl_2$	1	0.83	1468.6	49.6
	2	0.75	1327.2	78.6
	3	0.91	1609.9	84.8

* Data point considered an outlier and not included in the average. ** Displacement data unavailable to estimate elastic modulus.

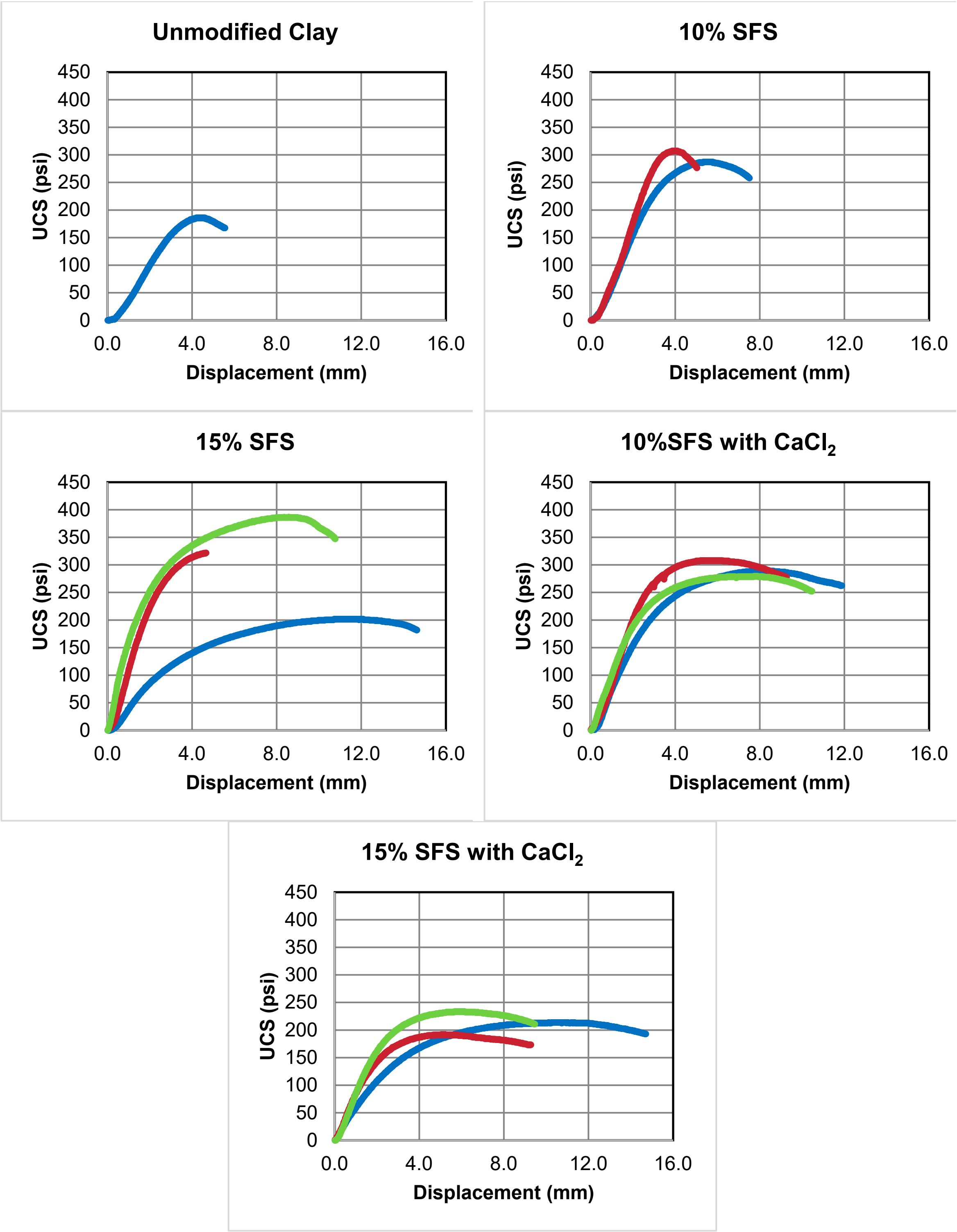

Figure S1. Stress-displacement curves for the five mixtures and replicates. While three replicate UCS tests were performed for each mix, the displacement data was not recorded for all tests.

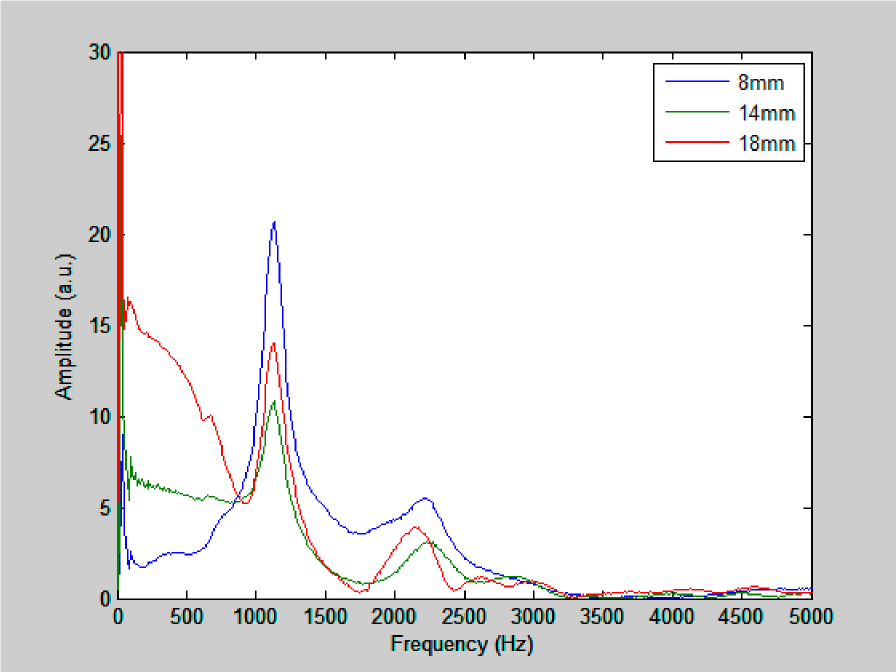

Figure S2. Longitudinal resonance for the un-stabilized clay for each impactor size.

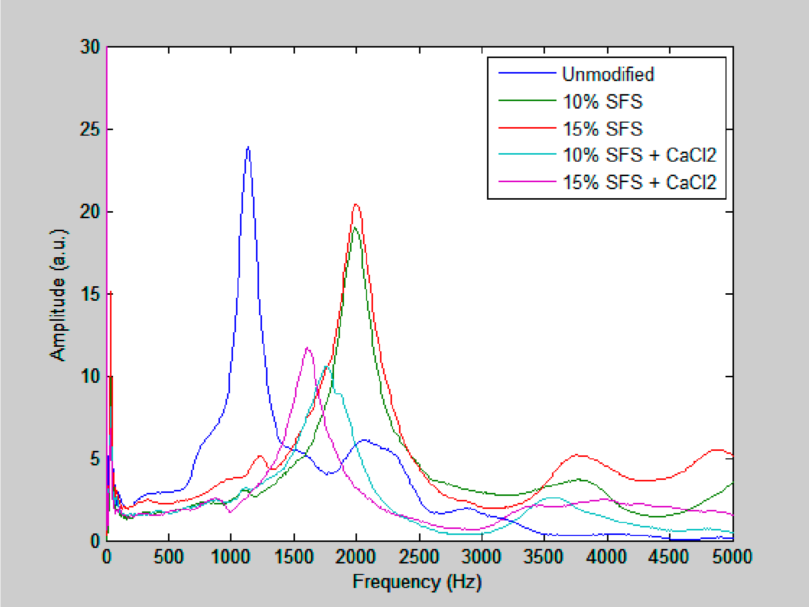

Figure S3. Comparison of the longitudinal resonant frequency responses generated with an 8-mm impactor for each mix.

Table S2. Replicate test data for longitudinal resonance frequency.

Sample	Test Replicate	Impactor Size	Mass (g)	Length (cm)	Diameter (cm)	Frequency (Hz)	Dynamic Modulus (MPa)
Unmodified Clay	1	8 mm	1127.91	14.2	7	1130	213
	2	8 mm				1140	216
	3	8 mm				1140	216
	1	14 mm				1130	213
	2	14 mm				1110	205
	3	14 mm				1100	201
	1	18 mm				1120	209
	2	18 mm				1140	216
	3	18 mm				1130	213
10% SFS	1	8 mm	1148.25	14.2	7	1990	671
	2	8 mm				1990	671
	3	8 mm				1990	671
	1	14 mm				1960	651
	2	14 mm				1940	638
	1	18 mm				1960	651
	2	18 mm				1950	644
	3	18 mm				1990	671
15% SFS	1	8 mm	1172.95	14.2	7	2000	692
	2	8 mm				2000	692
	3	8 mm				1990	686
	1	14 mm				2040	720
	2	14 mm				2010	699
	1	18 mm				1990	686
	2	18 mm				2040	720
10% SFS + $CaCl_2$	1	8 mm	1138.25	14.1	7	1750	511
	2	8 mm				1760	517
	3	8 mm				1870	583
	1	14 mm				1760	517
	2	14 mm				1770	523
	3	14 mm				1850	571
	1	18 mm				1750	511
	2	18 mm				1750	511
	3	18 mm				1760	517
15% SFS + $CaCl_2$	1	8 mm	1162.28	14.1	7	1610	442
	2	8 mm				1610	442
	1	14 mm				1630	453

	2	14 mm	1620	447
	1	18 mm	1600	436
	2	18 mm	1580	425
	3	18 mm	1600	436

Table S3. Replicate test data for transverse resonance frequency.

Sample	Test Replicate	Impactor Size	Frequency (Hz)
Unmodified Clay	1	8 mm	660
	2	8 mm	670
	3	8 mm	620
	1	14 mm	660
	2	14 mm	630
	3	14 mm	650
	1	18 mm	630
	2	18 mm	620
	3	18 mm	620

Table S4. Moisture-density data from Proctor tests.

Sample	Moisture Content	Dry Density (kg/m^3)
Unmodified Clay	8.8%	1777.1
	9.6%	1755.6
	11.0%	1766.7
	13.9%	1780.9
	15.4%	1772.0
	16.2%	1772.0
	19.1%	1734.4
	21.6%	1619.8
	23.5%	1561.7
10% SFS	11.8%	1776.0
	14.0%	1796.4
	15.4%	1812.9
	16.0%	1784.3
	17.7%	1750.9
	19.2%	1718.6
15% SFS	9.6%	1782.3
	11.2%	1804.1
	14.0%	1809.2
	16.0%	1808.7
	17.5%	1765.7
	19.7%	1721.2

© 2020 by the authors. Submitted for possible open access publication under the terms and conditions of the Creative Commons Attribution (CC BY) license (http://creativecommons.org/licenses/by/4.0/).
